# Study of Programmed Death Ligand 1 and EGFR/HER2 Expression in Non-Small-Cell Lung Carcinoma With a Clinicopathological Spectrum

**DOI:** 10.7759/cureus.16195

**Published:** 2021-07-05

**Authors:** Devina Laishram, Vandana Raphael, Evarisalin Marbaniang, Caleb Harris, Vikas Jagtap, Baphiralyne Wankhar

**Affiliations:** 1 Pathology, North Eastern Indira Gandhi Regional Institute of Health and Medical Sciences, Shillong, IND; 2 Surgical Oncology, North Eastern Indira Gandhi Regional Institute of Health and Medical Sciences, Shillong, IND; 3 Radiation Oncology, North Eastern Indira Gandhi Regional Institute of Health and Medical Sciences, Shillong, IND; 4 Radiology, North Eastern Indira Gandhi Regional Institute of Health and Medical Sciences, Shillong, IND

**Keywords:** non small cell lung carcinoma, programmed death ligand-1, epidermal growth factor receptor, her-2-neu, immunohistochemistry

## Abstract

Non-small-cell lung carcinoma (NSCLC) is a disease characterized by the upregulation of programmed death ligand 1 (PD-L1) along with alterations in epidermal growth factor receptor (EGFR) and HER2-neu (HER2) amplification in addition to EGFR mutation. In the present study, the expression of PD-L1 and EGFR and HER2-neu in NSCLC was studied and their expression in relation to various clinicopathological parameters was analysed. We studied 49 core biopsy specimens of NSCLC for PD-L1, EGFR and HER2-neu expressions using immunohistochemistry. Scoring was based on the intensity and percentage of tumour cells expressing the immunomarkers. PD-L1, EGFR and HER2-neu expression was seen in 20.4%, 32.7% and 14.2% of NSCLC, respectively. The analysis showed no significant difference in PD-L1 expression in relation to any clinicopathological parameters. Low or negative EGFR expression was significantly associated with positive lymph node status (P=0.04). HER2-neu expression showed a significant difference in relation to tumour histology (adenocarcinoma; P=0.01). Also, there was no difference noted with PD-L1 expression in relation to EGFR and HER2-neu expression. As our study has a small number of cases, the validation of the predictive and prognostic value of these markers in lung cancer patients requires further studies.

## Introduction

Globally, lung cancer is the most commonly diagnosed cancer accounting for 11.6% of the total cases and being the main cause of cancer death constituting 18.4% of the total cancer deaths [1]. It is also the commonest cancer in males in India accounting for 11.3% of all newly detected cancers and being the commonest cause of cancer death (13.7%) [2]. Although a good deal of research has been done on the subject, prognosis of this cancer remains poor with patient survival riding primarily on early detection and diagnosis. Recognizing characteristic genetic anomaly for potential target therapies may be a way forward [3]. Programmed death ligand 1 (PD-L1), the main ligand of programmed death 1 (PD-1) is upregulated in a subset of non-small-cell lung carcinoma (NSCLC) [4]. A distinguishing feature of NSCLC is the molecular mutation subsets in the epidermal growth factor receptor (EGFR), which is a major oncogenic driver in this cancer [5]. Another notable oncogene addiction in NSCLC is the human epidermal growth factor receptor 2 (HER2-neu) [5]. Having said that, the interrelation of these driver mutations with PD-L1 expression in NSCLC is still uncertain [4]. PD-1 and its ligand, PD-L1 play a major role in evading the tumour immune response and the formation of tumour microenvironment that contributes to tumour generation and development [6]. Some NSCLCs are characterised by activating mutations in the EGFR gene resulting in constitutive tyrosine kinase activity [7]. Unregulated activation inhibits tumour cell apoptosis and contributes to tumour progression [8]. The prognostic role of HER2-neu expression in lung cancer is still controversial. However, some studies have suggested that HER2-neu overexpression in NSCLC is a weak prognostic factor [9].

This study was undertaken with the aim of studying the expression and the interplay of these three markers, PD-L1, EGFR and HER2-neu, in NSCLC in the Northeastern region of India and to know their potential role as predictive biomarkers.

## Materials and methods

The present study was conducted in the Department of Pathology, North Eastern Indira Gandhi Regional Institute of Health and Medical Sciences, Shillong, India, from January 2018 to July 2020.The relevant clinical and radiological data were obtained for all the cases diagnosed by histopathological examination as non-small-cell lung cancer. Tumours were histologically verified. The cases with prior neoadjuvant therapy were excluded. Approval for the study was granted by the Institute Research and Ethics Committee (NEIG/IEC/M7/T12/19). American Joint Committee on Cancer (AJCC) TNM classification guidelines were used for clinical staging of the tumour. The assessment of PD-L1, EGFR and HER2-neu was done using immunohistochemistry (IHC) by the horse radish peroxidase (HRP) method. The paraffin-embedded sections of the tumour were stained by a monoclonal antibody raised against PD-L1 (clone Cal10; Master Diagnostica, Spain), HER2-neu (Dako, Denmark) and EGFR (clone EP22; Master Diagnostica).

Each slide was examined by two pathologists independently under a light microscope. They were blinded of the clinical history of the cases. For internal quality control and standardization of the process, sections of oral squamous cell carcinomas (SCCs), breast carcinomas and gastric carcinomas were used as positive controls and sections with absent primary antibody as negative controls. According to the intensity of staining of neoplastic cells and the percentage of positive cells, the results of immunostaining in tissues were scored. The cut-off for PD-L1 positive stained tumour is membranous staining of 1% of tumour cells; the expression <1% was evaluated as negative [10,11]. High and low scores of EGFR expression were defined using an H-score of 200 as the threshold. H-score was defined as a continuous variable with a scale ranging from 0 to 300 and was calculated using the following formula: 1 × (Percentage of weakly stained cells, 1+) + 2 × (Percentage of moderately stained cells staining, 2+) + 3 × (Percentage of strongly stained cells, 3+). Strong staining (3+) was clearly visible membranous staining using a ×4 objective lens; moderate staining (2+) required a ×10 or ×20 objective lens for clear observation and weak staining (1+) required a ×40 objective lens [12-13]. For HER2-neu, only membrane staining with an intensity of 3+ on a 0 to 3+ scale was considered positive.

The association between PD-L1, EGFR and HER2-neu expression with clinicopathological parameters was evaluated by chi-square (χ^2^) test and Fischer’s exact test. Probability (P) value of less than 0.05 was considered statistically significant.

## Results

**Figure 1:**
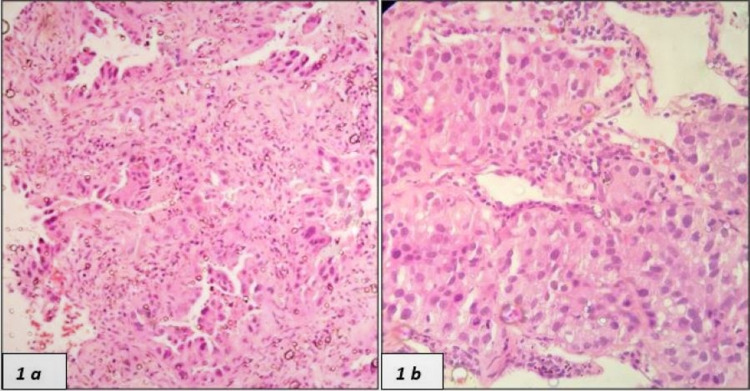
(1a) Adenocarcinoma (H&E, 100x) and (1b) squamous cell carcinoma (H&E, 100x)

A total of 49 cases fulfilling our inclusion and exclusion criteria were taken for analysis. All were core biopsy specimens. The age range was 33 to 80 years and the mean age was 61.8±10.8 years. A total of 39 patients were males (M) and 10 patients were females (F) with a M:F ratio of 4:1. Majority of the cases (n=23) were Khasis, followed by Assamese (n=13), Tripuri (n=5), Mizo (n=4), Arunachali (n=2) and Manipuri and Naga (n=1 each). Squamous cell carcinoma (SCC) constituted 35 cases and adenocarcinoma (ADC) constituted 14 cases (Figures 1a, 1b). According to the AJCC tumour staging classification, only 2 cases were of cT1, 9 cases were of cT2, 13 cases of cT3 and 25 were cases of cT4 stage. Nodal staging of the tumours included 9 cases of cN0, 10 cases of cN1, 21 cases of cN2 and 9 cases of cN3. There were 30 cases that had distant metastasis (M1) and rest 19 cases had no distant metastasis (M0). Only 2 cases were included in Stage I, 0 cases in stage II, 17 cases in Stage III and Stage IV had 30 cases.

**Figure 2:**
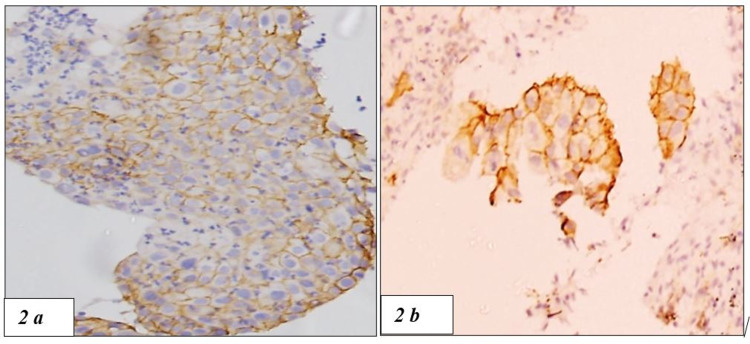
(2a and 2b) PD-L1 IHC showing membranous staining of the tumour cells (100x) PD-L1, programmed death ligand 1; IHC, immunochemistry

**Figure 3:**
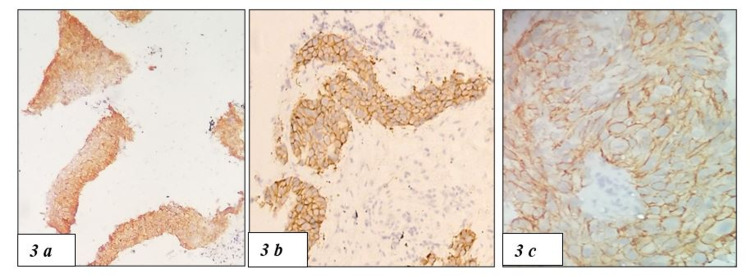
(3a-3c) EGFR IHC showing membranous positivity of the tumour cells at 40x ,100x and 400x, respectively EGFR, epidermal growth factor receptor; IHC, immunohistochemistry

**Figure 4:**
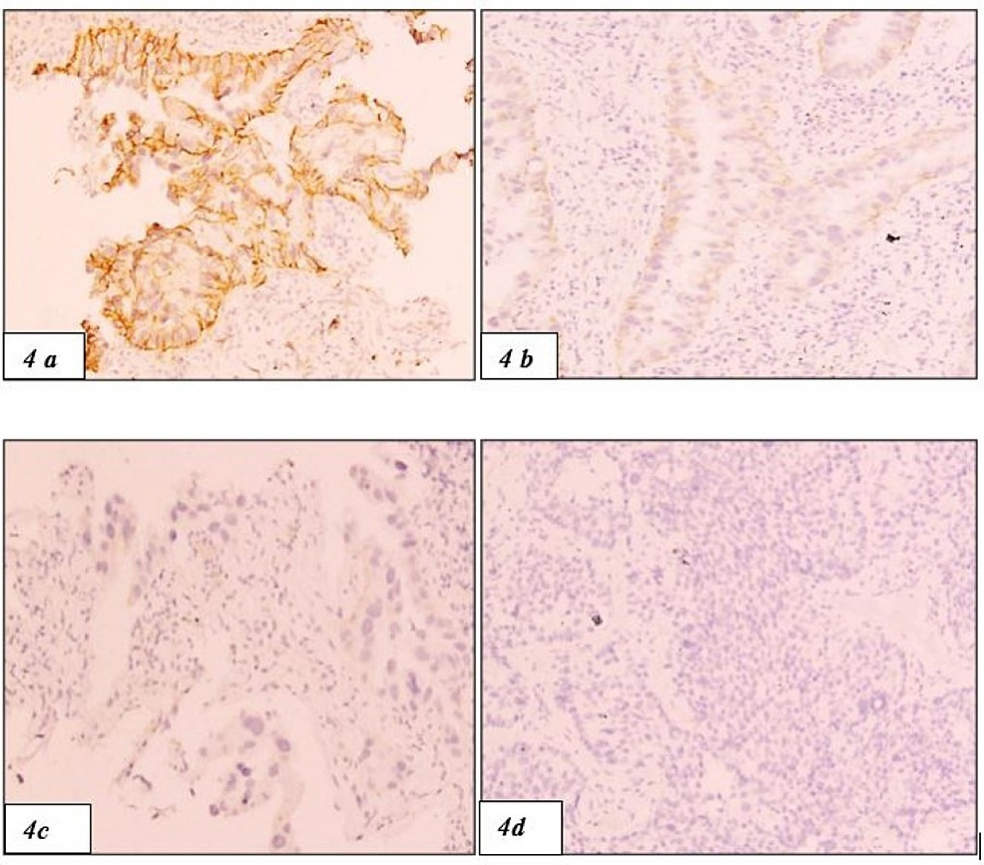
(4a-4d) HER2-neu IHC showing membranous staining of the tumour cells with scores 3+, 2+, 1+ and 0, respectively (100x) HER2, human epidermal growth factor receptor 2; IHC, immunohistochemistry

PD-L1 positivity was seen in 10 out of 49 cases (Figures 2a, 2b). High EGFR expression was seen in 16 cases (Figures 3a-3c) and the rest 33 cases were negative or low for EGFR expression. HER2-neu expression was seen in 7 cases of NSCLC and the rest 42 cases were equivocal (n=4) or negative (n= 38) for HER2-neu (Figures 4a-4d).

The association of PD-L1, EGFR and HER2-neu expression with age, gender, smoking, tumour histology, grading and TNM staging is described in Table 1.

**Table 1:** Associations of clinicopathological variables with EGFR, PD-L1 and HER2-neu in NSCLC EGFR, epidermal growth factor receptor; HER2, human epidermal growth factor receptor 2; PD-L1, programmed death ligand 1; NSCLC, non-small-cell lung carcinoma; ADC, adenocarcinoma; SCC, squamous cell carcinoma

Clinicopathological parameters		No. of cases	EGFR expression			PD-L1 expression			HER2-neu expression		
			Low (<200)	High (≥200)	P-value	Negative	Positive	P-value	Negative	Positive	P-value
Age (years)	<65	30	20	10	0.8	25	05	0.4	24	06	0.1
	≥65	19	13	06		14	05		18	01	
Gender	Male	39	26	13		32	07	0.4	35	04	0.1
	Female	10	07	03		07	03		07	03	
Smoking	Never	12	08	04	01	09	03	0.6	09	03	0.3
	Current/former	37	25	12		30	07		33	04	
Histology	SCC	35	25	10	0.3	27	08	0.7	33	02	0.01
	ADC	14	08	06		12	02		09	05	
Grade	G1	03	03	0	0.5	03	0	0.3	03	0	0.6
	G2-G3	46	30	16		36	10		39	07	
Tumour size (mm)	cT1	02	02	0	01	02	0	1	02	0	01
	cT2-T4	47	31	16		37	10		40	07	
Lymph node metastasis	cN0	09	03	06	0.04	05	04	0.07	09	0	0.3
	cN1-N3	40	30	10		34	06		33	07	
Metastasis	M0	19	12	07	0.7	25	4	0.1	18	01	0.2
	M1	30	21	09		14	6		24	06	
Staging	I	02	01	01	01	02	0	0.4	02	0	01
	II-IV	47	32	15		37	10		40	07	

PD-L1 expression was correlated with EGFR and HER2-neu expression and EGFR expression was correlated with HER2-neu expression, which is enumerated in Tables 2, 3, 4, respectively.

**Table 2:** Association of PD-L1 expression with EGFR expression in NSCLC EGFR, epidermal growth factor receptor; PD-L1, programmed death ligand-1; NSCLC, non-small-cell lung carcinoma

	EGFR low expression	EGFR high expression	P-value
PD-L1 negative	22	17	0.1
PD-L1 positive	03	07	

**Table 3:** Association of PD-L1 expression with HER2-neu expression in NSCLC HER2, human epidermal growth factor receptor 2; PD-L1, programmed death ligand-1; NSCLC, non-small-cell lung carcinoma

	HER2-neu negative	HER2-neu positive	P-value
PD-L1 negative	32	07	0.3
PD-L1 positive	10	0	

**Table 4:** Association of HER2-neu expression with EGFR expression in NSCLC EGFR, epidermal growth factor receptor; HER2, human epidermal growth factor receptor 2; NSCLC, non-small-cell lung carcinoma

	EGFR low expression	EGFR high expression	P-value
HER2-neu negative	29	13	0.6
HER2-neu positive	04	03	

## Discussion

For the purpose of studying the expression of PD-L1, EGFR and HER2-neu expression, we included 49 cases of NSCLC that fulfilled the inclusion criteria with 35 cases (71.4%) of SCCs and 14 cases (28.5%) of ADCs and analysed the pattern of expression with various clinicopathological parameters.

The prevalence of PD-L1 expression in NSCLC ranges from 24% to 60%, even with a cut-off for positivity set at 5% [14]. In the present study, PD-L1 positivity was seen in 20.4% (10 cases) of NSCLC cases. Schmidt et al. found that PD-L1 was expressed by 24% of the NSCLC samples, which is similar to our study [15]. Okita et al. observed that PD-L1 was overexpressed in 14% of the NSCLC cases [16].

Preceding studies have found PD-L1 expression to be associated with poor prognosis. However, despite extensive research, its predictive and prognostic value in lung cancer remains irresolute reflecting the conflicting results of previous studies [4].

Schmidt et al. found PD-L1 expression in NSCLC to be associated with improved prognosis, tumour histology, increased tumour size and lymph node status [14]. Okita et al. observed that PD-L1 overexpression was correlated with gender, smoking status, histology, histologic grade, lymph node metastasis, and pathological stage [16]. A meta-analysis done by Zhang et al. showed that the expression of PD-L1 was increased in those with male gender, positive smoking history, SCC histology, high histological grade, larger tumour size, positive nodal metastasis status, and an advanced clinical stage [4].

In the present study, no statistically significant difference in PD-L1 expression in relation to parameters, such as age, gender, smoking history, tumour histology, tumour grade, tumour size, nodal metastasis status and tumour stage, was noted. Most of the studies had used 91 to 321 cases whereas our study had only 49 cases that could explain this difference in our observation when compared to these studies. It may also be noted that the different thresholds to define positive expression and different sets of baseline characteristics interfere in the comparison of different studies showing correlation of PD-L1 expression in NSCLC. Standardized methods and thresholds of PD-L1 positivity are clearly needed to promote studies on PD-L1 as a prognostic biomarker.

HER2-neu amplification in NSCLC has been known to be associated with female gender, never-smoking status, adenocarcinoma histology and poor prognosis [17]. Similarly, seven cases (14.3%) of NSCLC showed staining score of 3 with IHC for HER2-neu with significant difference (P=0.01) in the expression between ADC (10.2%) and SCC (4%) in the present study. Auliac et al. found HER2-neu mutations to be more common in younger patients, female patients, non-smokers, and ADCs [18]. A European cohort also demonstrated HER2-neu mutations to be associated with female gender and a never-smoking status and similar observations were also made in an Asian analysis [18]. Takenaka et al. found HER2-neu overexpression to be associated with unfavourable prognosis [5]. Okita et al. found that HER2-neu overexpression was correlated with gender, smoking status, tumour size, histology, histologic grade, pleural invasion, vascular invasion and tumour stage [16]. On the contrary, Pugh et al. showed that HER2-neu positivity on fluorescence in situ hybridization was not associated with EGFR mutations or amplifications, gender, ethnicity, smoking status, adenocarcinoma histology and gefitinib activity [19]. Similarly, we did not find any statistically significant difference in HER2-neu immunoexpression in relation to parameters like age, gender, smoking status, tumour grade, tumour size, nodal status and stage of tumour in our study.

It should be noted that the disparities in the overexpression of HER2-neu are most probably due to variations in patient populations studied and the methods used. Although IHC was the most commonly used method to detect HER2-neu overexpression in most of the studies, the IHC results can vary according to the primary antibody used, antibody dilution, type of tissue sample ( paraffin-embedded or frozen sections), the threshold used for establishing HER2-neu positivity and whether membrane or cytoplasmic immunostain is assessed. Less number of samples and the heterogenous population studied might explain the variable results in our study and the number of positive cases is too small for any conclusive evidence.

EGFR overexpression or mutations in NSCLC have been observed in 43%-89% of cases [20]. More than 90% of the recognised EGFR mutations in the tyrosine kinase domain occur as short in-frame deletions in exon 19 or as point mutations in exon 21 [20]. Li et al. found that EGFR overexpression was seen in 48% of NSCLC cases that correlated with EGFR gene amplification. However, the EGFR IHC results showed no significant interrelation with EGFR mutation status [21]. Thus, EGFR overexpression by IHC shows an association with EGFR amplification but is not of value in predicting the presence of EGFR mutations that implies that IHC negativity in tumours will not exclude the possibility of detecting mutation by gene amplification.

Some studies have shown some significant difference in the expression in relation to several clinicopathological parameters. Ohsaki et al. found that more EGFR positive cases were observed in SCCs and had shorter survival periods [22]. Okita et al. found that EGFR overexpression was correlated with histology, histologic grade, lymphatic invasion, vascular invasion and lymph node metastasis [16].

In the present study, EGFR overexpression was seen in 32.7% (16 cases) of NSCLC cases and the rest 67.3% (33 cases) had low or negative expression for EGFR. No significant difference in EGFR expression was noted in our study in relation to parameters like age, gender, smoking status, tumour grade, tumour size, tumour histology and stage of tumour; however, low or negative EGFR expression was noted with positive lymph node status (P=0.04). Similarly, Selvaggi et al. found no correlation between EGFR overexpression and histology, but found a higher EGFR expression in stage III when compared to earlier stages [23]. Avilés-Salas et al. found that EGFR is overexpressed in >60% of NSCLC cases. Furthermore, no differences were observed in the patient's age, smoking history, histological type and disease stage when patients with an EGFR score of <200 were compared with those with a score ≥200, which is similar to our study [12].

Several studies have shown EGFR mutation status to be associated with PD-L1 expression [24]. However, the mechanism through which EGFR activation influences PD-L1 expression and the potential role of blocking PD-1/PD-L1 in EGFR-mutant NSCLC treated with EGFR tyrosine kinase inhibitors (TKIs) are largely unknown [25].

A study by Chen et al. demonstrated that PD-L1 expression in EGFR-mutant NSCLC cell lines was higher when compared to wild-type EGFR cell lines [25]. A meta-analysis by Zhang et al. showed that high PD-L1 expression was associated with EGFR mutations. However, some studies have shown that PD-L1 expression was associated with EGFR wild type, while other studies have shown no association between PD-L1 expression and EGFR mutations [4]. Azuma et al. described a significant and an independent association of EGFR mutations and adenocarcinoma histology with increased PD-L1 expression [26]. Okita et al. found that PD-L1 overexpression is linked with poor prognosis and is associated with EGFR expression but showed an inverse relationship with HER2-neu expression in NSCLC [16].

However, we found no significant correlation between PD-L1 positivity and EGFR or HER2-neu overexpression. The discrepancies among different studies might reflect the heterogeneous nature of the study population and variable threshold of PD-L1 expression and use of IHC over genetic mutation analysis of EGFR. In our study, we used IHC on core needle biopsy instead of genetic mutation test for expression of EGFR that was also a major limitation.

Most of the cases were lost to follow-up and we could not correlate the relationship of these markers with management modality and overall survival, and thus, additional information on their role as the predictive biomarker of tumour behaviour could not be ascertained from this study.

## Conclusions

Squamous cell carcinoma of the lung is more common than adenocarcinoma in this part of our country with a male predominance and presenting with cough, dyspnoea and chest pain and less frequently with haemoptysis. HER2-neu expression was significantly seen higher with adenocarcinoma than with squamous cell carcinoma. There was no difference noted with PD-L1 and EGFR/HER2-neu immunoexpression. Hence, the validation of the prognostic value of PD-L1 or EGFR/HER2 immunoexpression in patients with lung cancer requires further studies with a larger sample size to establish their role in identifying the molecular subgroup for the purpose of target therapy and overall survival.
